# Supplementary material for: Balance between asymmetry and abundance in multi-domain DNA-binding proteins may regulate the kinetics of their binding to DNA
Source: PLoS Comput Biol. 2020 May 26;16(5):e1007867. doi: 10.1371/journal.pcbi.1007867 (PMC7274453; doi:10.1371/journal.pcbi.1007867)
Supplement: S1 Table — (DOCX) [file pcbi.1007867.s001.docx]

| #ZFs in ZFP | #Cases in the dataset | Uniprot Id |
| --- | --- | --- |
| 3 | 37 | O14901,O15209,O43298,O43474,O95600,P08047,P11161,P18146,  P57682,Q02086,Q02446,Q02447,Q13118,Q13351,Q13886,Q13887,  Q15697,Q5JT82,Q6ZN18,Q86T24,Q8IXZ3,Q8NB15,Q8NCN2,Q8NDX6,  Q8TDD2,Q8WW38,Q96IQ9,Q96N95,Q99612,Q9BXK1,Q9H4Q4,Q9UIH9,  Q9UK33,Q9Y2Y4,Q9Y2Y9,Q9Y4X4,Q9Y5W3 |
| 4 | 22 | O15156,O75626,O95365,O95863,P17028,P19544,P25490,Q15916,  Q86UQ0,Q8NAM6,Q8NC26,Q8NCP5,Q96C55,Q96MM3,Q96T25,Q9H4T2,  Q9HCK0,Q9NQX0,Q9P0T4,Q9UQR1,Q9Y2X9,Q9Y330 |
| 5 | 24 | O43623,O43829,O60481,O95409,P08151,P10070,P10071,P17022,  P17026,Q15915,Q3MJ62,Q6N043,Q86VK4,Q8N2R0,Q8N554,Q96CK0,  Q96GC6,Q96JP5,Q9BU19,Q9BUG6,Q9BWW7,Q9BZE0,Q9UDV7,Q9UNY5 |
| 6 | 15 | A0PJY2,P17029,P41182,P52739,Q14872,Q5T0B9,Q5VTD9,Q63HK3,  Q86XF7,Q8IWY8,Q96LW9,Q99684,Q9GZV8,Q9NX65,Q9UPG8 |
| 7 | 18 | A8K8V0,P10075,P17041,P36508,P51504,P52747,Q53GI3,Q5VV52,  Q6DJT9,Q6NSZ9,Q6P9G9,Q6ZSB9,Q86W11,Q96C28,Q96H86,Q9NR11,  Q9UM63,Q9Y5A6 |
| 8 | 12 | O43167,O95201,P10073,P17036,Q15973,Q6P2D0,Q7L3S4,Q86TJ5,  Q8TF68,Q96EG3,Q9GZX5,Q9NPC7 |
| 9 | 19 | Q05516,Q08AG5,Q15776,Q16600,Q6AW86,Q8IYX0,Q8N3J9,Q8NF99,  Q8TD17,Q92664,Q96I27,Q96NG5,Q9BSG1,Q9H7X3,Q9NV72,Q9NW07,  Q9UFB7,Q9UJL9,Q9Y2D9 |
| 10 | 13 | P98168,Q15072,Q2QGD7,Q5HY98,Q5T619,Q6AZW8,Q9BS31,Q9H116,  Q9H5H4,Q9H9D4,Q9HBT8,Q9UIE0,Q9UK11 |
| 11 | 17 | O43309,P10074,P13682,P49711,Q14592,Q15928,Q15937,Q2M3W8,  Q7L2R6,Q86Y25,Q8N1W2,Q8N895,Q8NDP4,Q8NEK5,Q8NI51,Q8TF39,  Q96HQ0 |
| 12 | 21 | A2RRD8,P51508,Q09FC8,Q12901,Q16587,Q6NX45,Q6P1L6,Q6P280,  Q6ZMS4,Q86UD4,Q8IYI8,Q8NEM1,Q8WV37,Q96CS4,Q96N58,Q96RE9,  Q9C0F3,Q9H8G1,Q9NQX6,Q9NUA8,Q9UJW8 |
| 13 | 19 | O43296,O75123,P08048,P15622,P17031,P28698,Q08ER8,Q14593,  Q14929,Q6NX49,Q6ZN57,Q8IW36,Q8N141,Q96NJ6,Q96PQ6,Q9NQZ8,  Q9P0L1,Q9ULM2,Q9Y2G7 |
| 14 | 7 | A6NHJ4,P52737,Q14584,Q6ZNH5,Q8TBZ5,Q96NL3,Q96SZ4 |
| 15 | 13 | P17014,P17019,P17024,P52736,Q494X3,Q6P9A3,Q8N184,Q8N7M2,  Q8NEP9,Q8NHY6,Q8TBZ8,Q8TD23,Q9Y473 |
