# Supplementary material for: Balance between asymmetry and abundance in multi-domain DNA-binding proteins may regulate the kinetics of their binding to DNA
Source: PLoS Comput Biol. 2020 May 26;16(5):e1007867. doi: 10.1371/journal.pcbi.1007867 (PMC7274453; doi:10.1371/journal.pcbi.1007867)
Supplement: S2 Table — Correlation coefficient between asymmetry and abundance. Data trend shows that asymmetry and abundance are better anti-correlated in non-specific interactions compared to specific interactions, though the correlation-coefficients are low. (DOCX) [file pcbi.1007867.s002.docx]

**ZF^3-6^**

**ZF^3^**

**ZF^4^**
