# Supplementary figures and images for: Balance between asymmetry and abundance in multi-domain DNA-binding proteins may regulate the kinetics of their binding to DNA
Source: PLoS Comput Biol. 2020 May 26;16(5):e1007867. doi: 10.1371/journal.pcbi.1007867 (PMC7274453; doi:10.1371/journal.pcbi.1007867)

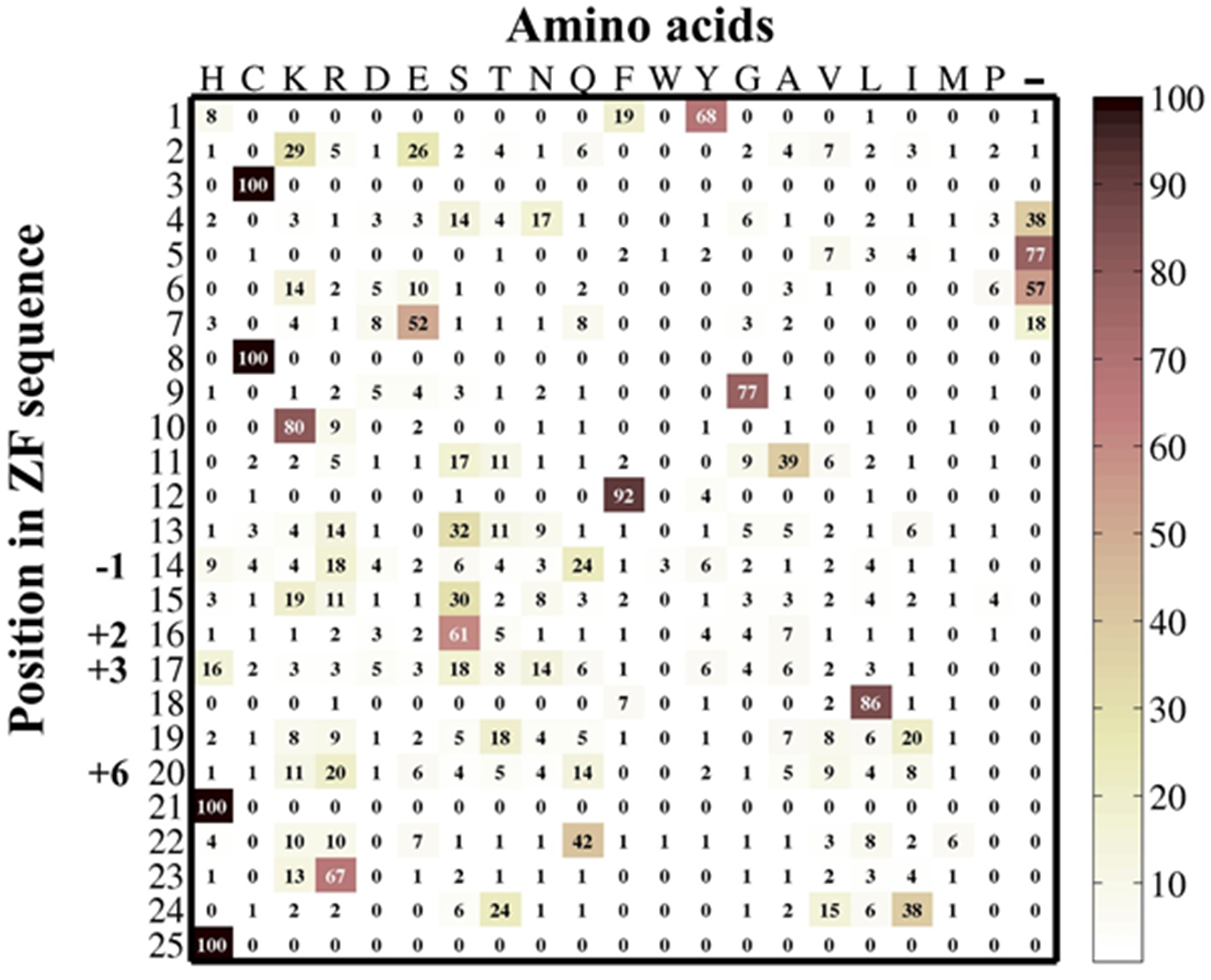

Supplement: S1 Fig — The propensity of each amino acid residue to locate at each position in the ZF sequence was calculated from multiple sequence alignment of 1911 C2H2-type ZFP sequences using the sequence of the first finger of the human early growth response protein 1 (Egr1, pdb id 4X9J) as a template. The last column corresponds to the probability of finding a gap at each position. (TIF) [file pcbi.1007867.s004.tif]

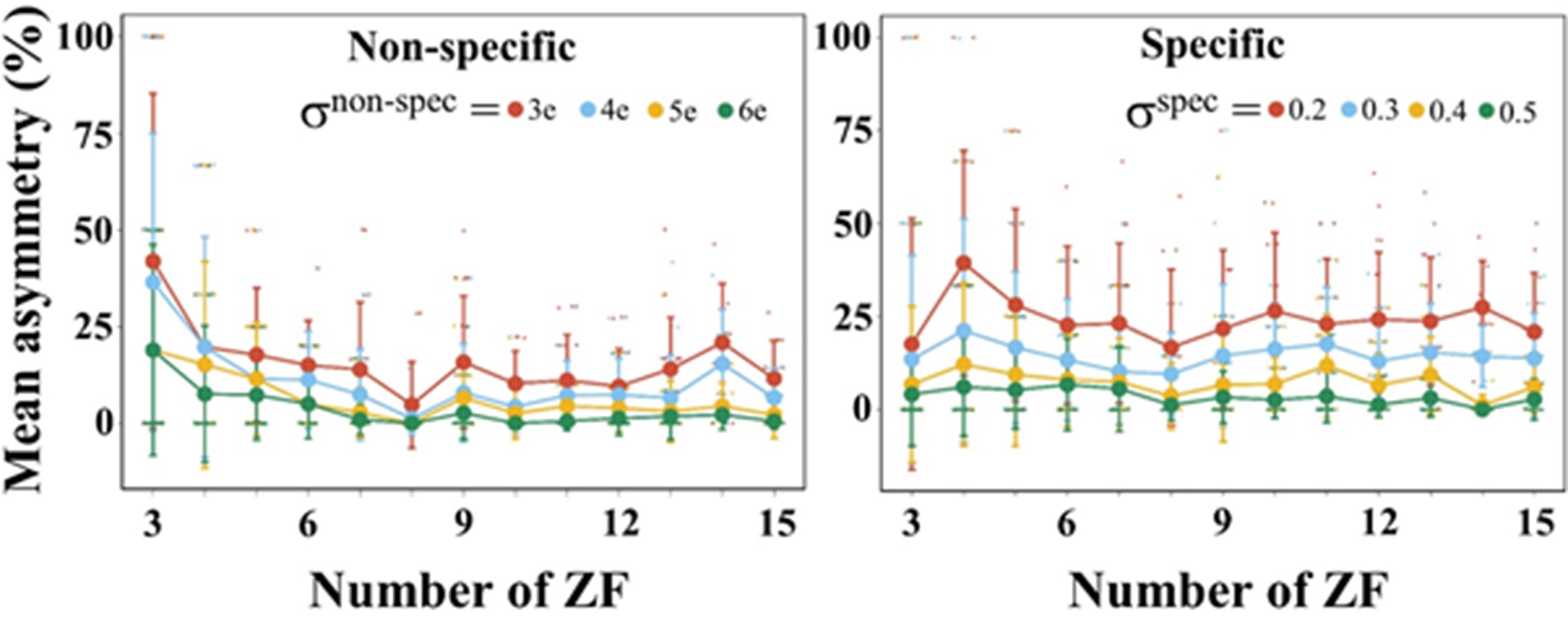

Supplement: S2 Fig — Plots similar to those in Fig 4 are shown for different σnonspec and σspec. (TIF) [file pcbi.1007867.s005.tif]

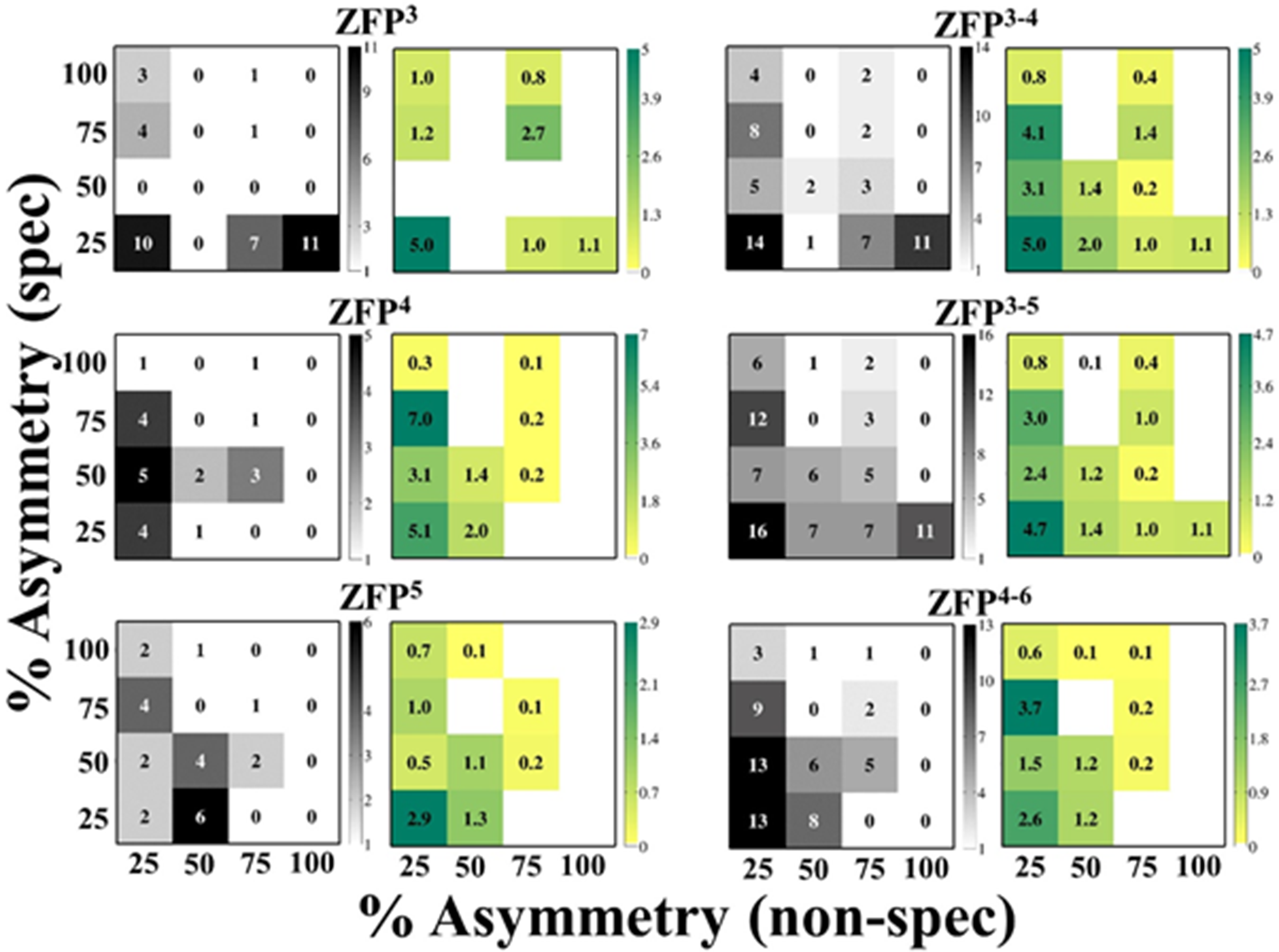

Supplement: S3 Fig — The presented analysis is for adjacent zinc finger domains in ZFPs comprising 3, 4, 5, 3–4, 3–5 and 4–6 zinc-finger domains (as indicated in the title of each plot). The analysed ZFPs were binned into 16 bins on the basis of the percentage of their non-specific and specific asymmetry scores. The number of ZFPs in each of the bins is shown with grey colour bar. The mean cellular abundances of all the ZFPs in each of the beans bins is shown with yellow-to-green colour bar. This analysis was performed using σnonspec = 3 and σspec = 0.2. (TIF) [file pcbi.1007867.s006.tif]

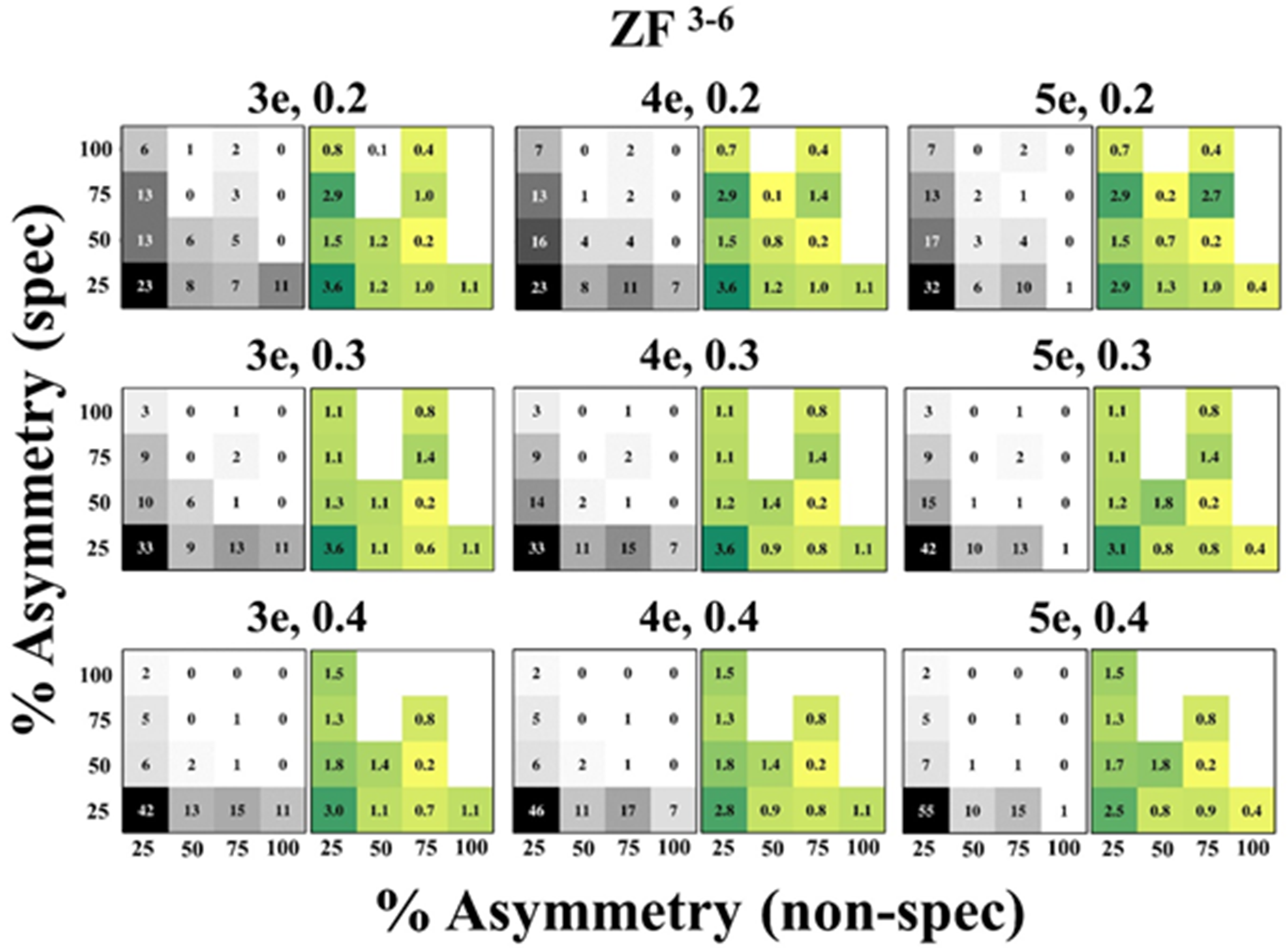

Supplement: S4 Fig — The net charge cut-off value used to define asymmetrical non-specific binding (σnonspec) and the binding specificity score used to define asymmetrical specific binding (σspec) are shown at the top of each panel. Plots similar to those in Fig 8 are shown for the dataset of 98 zinc finger proteins containing 3–6 zinc finger domains. The number of ZFPs in each of the bins is shown with grey colour bar. The mean cellular abundances of all the ZFPs in each of the beans bins is shown with yellow-to-green colour bar. (TIF) [file pcbi.1007867.s007.tif]
