# Supplementary material for: Balance between asymmetry and abundance in multi-domain DNA-binding proteins may regulate the kinetics of their binding to DNA
Source: PLoS Comput Biol. 2020 May 26;16(5):e1007867. doi: 10.1371/journal.pcbi.1007867 (PMC7274453; doi:10.1371/journal.pcbi.1007867)

מכון ויצמן למדע
WEIZMANN INSTITUTE OF SCIENCE

Prof. Yaakov (Koby) Levy
Dept. of Structural Biology
Weizmann Institute of Science,
Rehovot, 76100, Israel

Phone: ++972-(0)8-934 4587
Fax: ++972-(0)8-934 4136

Koby.Levy@weizmann.ac.il

April 1, 2020

PLoS Computational Biology

Dear Prof. Chen
Dear Prof. Ben Tal

Thank you very much for handling our manuscript (PCOMPBIOL-D-19-02058). Enclosed please find our revised version for the manuscript entitled "Balance between asymmetry and abundance in multi-domain DNA-binding proteins may regulate the kinetics of their binding to DNA" by Arumay Pal and Yaakov Levy.

We are very grateful for the editors and the two reviewers for examining our manuscript thoroughly, and adding constructive comments. We have revised our manuscript and the Supporting Information. The revision includes changes in the text, adding one figure (Figure 10) and two tables (Tables 2 and 3) to the Supporting Information. Following is a detailed description of the changes made in the manuscript.

Reviewer #1:

In this manuscript, Pal and Levy describe a statistical analysis of Cys2-His2-type zinc fingers (ZFs) of human transcription factors. In their previous studies, the Levy group conducted coarse-grained simulations of DNA search by ZF proteins and many other classes of transcription factors and found that asymmetry in binding affinity among DNA-binding domains in the same protein might be important for the search kinetics. Based upon the previous findings, the authors examined whether the importance of the asymmetry in the search kinetics is supported by statistical data of human ZF proteins. The authors assessed the asymmetry based on the overall charge distribution among ZFs and the amino-acid types of the key residues for the sequence-specific binding to DNA. Interestingly, ZF proteins with low asymmetry tend to be abundant. Since rapid search kinetics would be nonessential for abundant proteins, the statistical anti-correlation between the asymmetry and abundance seems to support the importance of the asymmetry in the search kinetics. I would like to recommend publication of this manuscript after minor revision in the following issues.

1. In my opinion, the most important result in this work is the data shown in Figure 8. However, the current presentation of this result seems too qualitative. More quantitative information

about the statistical significance of the anti-correlation between the asymmetry and the abundance is desirable.

We thank the reviewer for the suggestion to highlight the statistical significance of the anti-correlation between the asymmetry and the abundance in Figure 8 which is indeed the most important result of this study. The correlation coefficient between asymmetry and abundance is low for non-specific interactions: -0.25, -0.40, -0.22 for ZFP^3, ZFP^4 and $ZFP^{3\text{-}6}$ dataset, respectively. The correlation is independent of the cut-off used to define non-specific asymmetry. However, asymmetry in specificity is less anti-correlated with abundance, though the correlation coefficients were found to be negative in all cases but the values are -0.08 (Table S2).

Further, we tested statistical significance by unpaired t-test to verify the difference of mean abundance between symmetric and asymmetric ZFPs. As done in Figure 7, the $ZFP^{3\text{-}6}$ dataset was divided into two groups depending on the percentage of asymmetric zinc finger pairs in each protein: symmetric zinc-finger proteins (containing <50% asymmetric pairs) and asymmetric zinc-finger proteins (containing ≥50% asymmetric pairs). This classification was performed separately for nonspecific binding (on the basis of electrostatic net charge) and specific binding (on the basis of the specificity score). In the case of non-specific binding, an unpaired t-test showed that the mean abundance of the symmetric group (2.33) is significantly higher than the asymmetric group mean (0.83) at 95% confidence level (p-value = 0.006). However, the mean abundance is not significantly different for specific binding, 1.92 and 1.85 (p-value = 0.943) for symmetric and asymmetric groups, respectively. A similar trend was observed when asymmetry was calculated with different non-specific and specific cut-offs (see Table S2).

These statistics are in agreement with our main findings that the ZFPs with higher non-specific symmetry are also more abundant in the cell, whereas ZFPs characterized by lower non-specific symmetry are less abundant in the cell, irrespective of their degree of specific symmetry. A discussion on this new analysis was added to the Results.

2. The PaxDb database provides abundance data for various cell types. Depending on cell types, the expression levels of individual ZF proteins could be quite different. Which cell types did the authors use for the PaxDb data? Do the authors get the same conclusion for different cell types?

Indeed, protein expression is dynamic where the results depend on the experimental design (growth conditions, quantification methods, type of cells, etc.). For a particular protein, PAXdb provides the abundance data along with the cell type, the experimental technique used and the source (literature), wherever available. However, for different ZFPs the abundance value is not available for all the cell types and obtained with all the experimental techniques. Hence, for each protein, we used the geometric mean of all the available abundance values for that particular protein, which is also provided by the database and is commonly used in statistical studies with multiple proteins.

3. It would be nice if the authors could present a graphical scheme to explain why the asymmetry and the abundance could be related in terms of search kinetics. Although there is a nice description in the text, a figure on this concept would be helpful for readers who are not familiar with the previous work of the Levy group.

Following the reviewer's suggestion, we added a schematic figure that describes the main conclusion of this study. This schematic figure appears as Figure 10.

Minor issues

In Page 3, 'FoKI' should be corrected to 'FokI'.

Corrected

Reviewer #2:

This is a very interesting and well-written article. It is essentially ready for publication, with the following minor optional comments which the authors can be trusted to implement if they wish to do so:

1) This work investigated the linkage between the degree of asymmetry in the tethered domains of multi-domain DNA-binding proteins and their cellular abundance. The dynamics of binding site search per se was not investigated. It is very plausible that the effect found here will indeed be related to the kinetics of binding and the dynamics of binding site search, but as of now this is just a hypothesis. I think it is a bit too much to mention kinetics in the title of the manuscript. I'd rather focus the title on the solid results obtained here.

We appreciate the reviewer's comment, yet the linkage we report here is supported experimentally for the Egr-1 zinc finger transcription factor. Indeed, additional experimental support is needed for the generalization. For that reason, this linkage between symmetry, abundance and recognition kinetics appears in the title as a proposal.

2) The terms such as non-specific asymmetry used in the abstract needs to be better explained because it is the central point of the article.

Following the reviewer's comment, the abstract was revised to clarify the meaning on asymmetry in zinc finger proteins.

3) Methods section: how many proteins were included in the final dataset with defined abundance and asymmetry?

The final dataset showing the correlation between abundance and asymmetry in Figure 8 consists of 98 $ZFP^{3\text{-}6}$s, the number is mentioned in the corresponding figure legend. Number of individual type proteins (ZFP^3, ZFP^4 etc.) is mentioned in Figure 3.

4) Methods section: I would add a dedicated paragraph explaining the asymmetry and all calculations of the specific and non-specific asymmetry. This explanation is provided later in the results section but it would be good to have it also in the Methods in a condensed form with equation.

Following the reviewer's comment, we added a description in the Methods section on the specific and non-specific asymmetry.

5) How was the threshold value selected for specific and non-specific asymmetry?

We used different cut offs to define non-specific and specific asymmetry to understand their effect (Figure S2-S4). Final threshold value was chosen as $\sigma^{non\text{-}spec} = 3e$ and $\sigma^{spec} = 0.2$ at which the symmetric and asymmetric groups had nearly equal number of proteins (Figure 7).

6) Figure 4: May be make violin plots rather than bar-plots, to show the distribution of values?

As suggested, we replaced the bar plots in Figure 4 by violin plots to show the distribution.

7) Figure 7: What is indicated by the dark rectangle?

Dark (black) and light (gray) rectangles corresponds to non-specific and specific scores, respectively, which is described at the top of the figure.

8) The protein abundance used throughput the article is cell-type specific. It is important to indicate the cell type. This may be mentioned in several places including figure legends, e.g. Figure 7.

This aspect was addressed in our response to the 2nd comment of reviewer #1.

Sincerely,
Yaakov (Koby) Levy

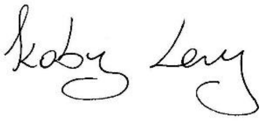

Supplement: Attachment — Submitted filename: Cover_letter_PlosCB_April1.pdf [file pcbi.1007867.s008.pdf]
